# Transcatheter aortic valve implantation in patients with chronic obstructive pulmonary disease

**DOI:** 10.1002/clc.23523

**Published:** 2020-12-01

**Authors:** Nauman Khalid, Sarah Aftab Ahmad, Nausharwan Butt, Waleed Tallat Kayani

**Affiliations:** ^1^ Section of Interventional Cardiology Saint Francis Medical Center Monroe Louisiana USA; ^2^ Section of Cardiothoracic Surgery Saint Francis Medical Center Monroe Louisiana USA; ^3^ Section of Internal Medicine MedStar Washington Hospital Center Washington District of Columbia USA; ^4^ Section of Interventional Cardiology, Baylor College of Medicine Houston Texas USA

Dear Editor,

Xiao et al evaluated the in‐hospital outcomes of transcatheter aortic valve implantation (TAVI) in patients with and without chronic obstructive pulmonary disease (COPD) by analyzing the National Inpatient Sample (NIS) database from 2011 through 2014.^1^ Results showed that patients with COPD had significantly increased risk of respiratory complications and pneumonia after TAVI but without significant increase in in‐hospital mortality, hospital length of stay, or nonrespiratory postoperative complications in comparison with the patients without COPD. We wanted to highlight a few points relevant to the article.

First, given the administrative nature of the NIS database, it lacks details regarding the severity of COPD quantified by the definitive criteria, and the co‐existence of pulmonary hypertension (PH), both of which are important factors for pre‐procedural risk stratification and planning. Patients with mild COPD constitute a markedly different group than oxygen‐dependent COPD. In particular, patients with very low forced expiratory volume in 1‐second (<30% predicted) are at an increased risk of poor outcomes following TAVI.^2^ PH presents as a common finding in patients undergoing TAVI. It has been shown that invasive stratification of PH according to hemodynamic profile predicts response to therapy as well as 1‐year mortality after TAVI.^3^ Second, the data regarding frailty, mobility, functional and nutritional status are not available in the NIS database, which are important considerations for clinical decision‐making among patients with severe aortic stenosis and COPD. Third, this study was conducted between 2011 and 2014, when TAVI was mainly performed under general anesthesia, whereas, conscious sedation has recently gained popularity and may be associated with fewer respiratory complications.^4^ Also, it is quite likely that the majority of these patients referred for TAVI were inoperable and high‐risk given the timeframe of this analysis.

Nonetheless, we would like to commend the authors for conducting this excellent study which supports the effectiveness of TAVI among patients with concomitant COPD despite the increased risk of respiratory complications.
